# Supplementary material for: Gene Co-Expression Analysis Reveals Functional Differences Between Early- and Late-Onset Alzheimer’s Disease
Source: Curr Issues Mol Biol. 2025 Mar 18;47(3):200. doi: 10.3390/cimb47030200 (PMC11941623; doi:10.3390/cimb47030200)

Supplementary Materials File S1

1. Study design flowchart:

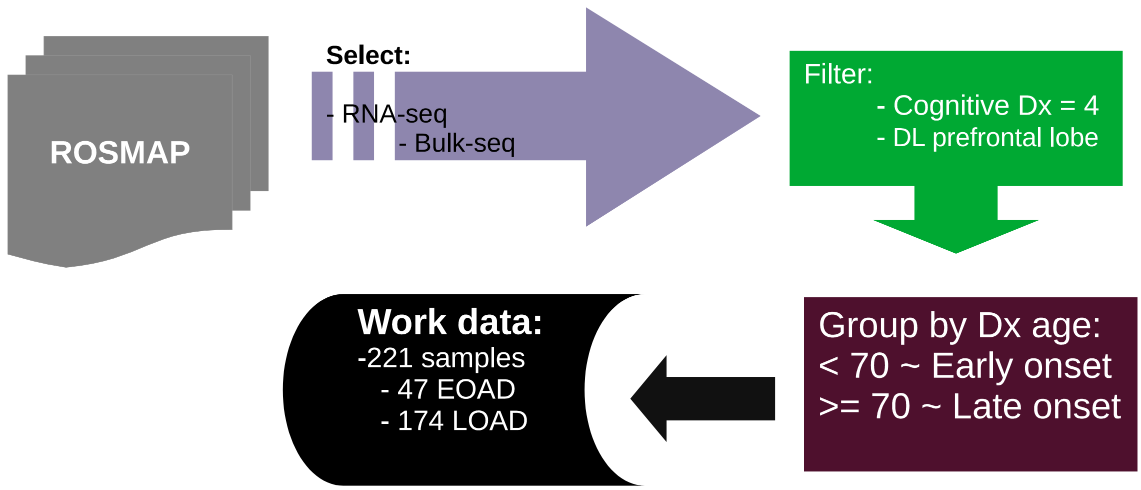

2. Age at diagnostic distribution for analysed samples:

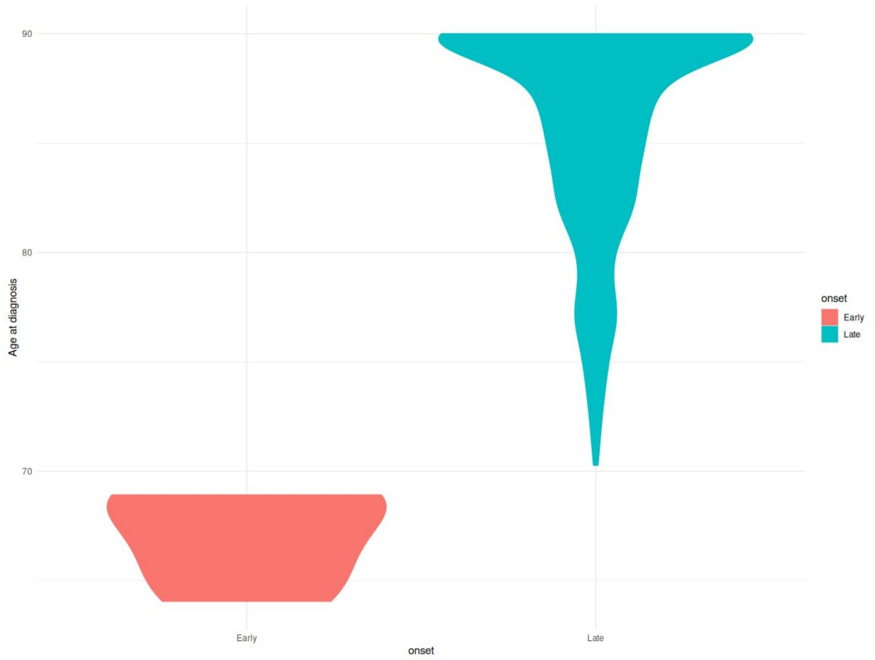

Supplement: Supplementary file 1 [file cimb-47-00200-s001.zip › cimb-3497031-supplementary.pdf]
